# Supplementary material for: Identifying Small‐Molecule Binding Sites for Epigenetic Proteins at Domain–Domain Interfaces
Source: ChemMedChem. 2018 Apr 17;13(10):1051–7. doi: 10.1002/cmdc.201800030 (PMC6001751; doi:10.1002/cmdc.201800030)
Supplement: Supplementary file 1 — Supplementary [file CMDC-13-1051-s001.pdf]

CHEMMEDCHEM
Chemistry & Drug Discovery

Supporting Information

Identifying Small-Molecule Binding Sites for Epigenetic Proteins at Domain–Domain Interfaces

David Bowkett+,[a] Romain Talon+,[a] Cynthia Tallant,[a] Chris Schofield,[b] Frank von Delft,[a, c, d] Stefan Knapp,[a, e] Gordon Bruton,[f] and Paul E. Brennan*[a, g]

cmdc_201800030_sm_Molecular_Formula_Strings.csv
